# Processing of Agilent microRNA array data

**DOI:** 10.1186/1756-0500-3-18

**Published:** 2010-01-22

**Authors:** Pedro López-Romero, Manuel A González, Sergio Callejas, Ana Dopazo, Rafael A Irizarry

**Affiliations:** 1 Department of Cardiovascular Epidemiology and Population Genetics, Centro Nacional de Investigaciones Cardiovasculares Carlos III (CNIC), Madrid, Spain; 2 Department of Epidemiology, Johns Hopkins Bloomberg School of Public Health, Baltimore, MD, USA; 3 Department of Regenerative Cardiology, Centro Nacional de Investigaciones Cardiovasculares Carlos III (CNIC), Madrid, Spain; 4 Genomics Unit, Centro Nacional de Investigaciones Cardiovasculares (CNIC), Madrid, Spain; 5 Department of Biostatistics, Johns Hopkins Bloomberg School of Public Health, Baltimore, MD, USA

## Abstract

**Background:**

The Agilent microRNA microarray platform interrogates each microRNA with several copies of distinct oligonucleotide probes and integrates the results into a total gene signal (TGS), using a proprietary algorithm that makes use of the background subtracted signal. The TGS can be normalized between arrays, and the Agilent recommendation is either not to normalize or to normalize to the 75^th ^percentile signal intensity. The *robust multiarray average algorithm *(RMA) is an alternative method, originally developed to obtain a summary measure of mRNA Affymetrix gene expression arrays by using a linear model that takes into account the probe affinity effect. The RMA method has been shown to improve the accuracy and precision of expression measurements relative to other competing methods. There is also evidence that it might be preferable to use non-corrected signals for the processing of microRNA data, rather than background-corrected signals. In this study we assess the use of the RMA method to obtain a summarized microRNA signal for the Agilent arrays.

**Findings:**

We have adapted the RMA method to obtain a processed signal for the Agilent arrays and have compared the RMA summarized signal to the TGS generated with the image analysis software provided by the vendor. We also compared the use of the RMA algorithm with uncorrected and background-corrected signals, and compared quantile normalization with the normalization method recommended by the vendor. The pre-processing methods were compared in terms of their ability to reduce the variability (increase precision) of the signals between biological replicates. Application of the RMA method to non-background corrected signals produced more precise signals than either the RMA-background-corrected signal or the quantile-normalized Agilent TGS. The Agilent TGS normalized to the 75% percentile showed more variation than the other measures.

**Conclusions:**

Used without background correction, a summarized signal that takes into account the probe effect might provide a more precise estimate of microRNA expression. The variability of quantile normalization was lower compared with the normalization method recommended by the vendor.

## Background

MicroRNAs are a family of small single-stranded non-coding RNAs which regulate gene expression [1]. Functional studies show that microRNAs participate in virtually every cellular process investigated, and changes in their expression might underlie many human pathologies [2]. The main research tool for identifying microRNAs involved in specific cellular processes is gene expression profiling using microarray technology. The microRNA Agilent microarrays [3] use different oligonucleotide probes for each individual microRNA that are replicated a number of times across the array surface. The Agilent Feature Extraction image analysis software (AFE) computes a summary measure for each microRNA, referred to as total gene signal (TGS), based on the robust average of all the background subtracted signals for each replicated probe. To make statistical inferences, Agilent recommends using either the non-normalized TGS or the TGS normalized to the 75^th ^percentile signal intensity, and several studies have pointed out that data normalization improves sensitivity and specificity over non-normalized microRNA data [4,5]. An alternative approach is to use the robust multiarray average (RMA) algorithm, developed by Irizarry et al. [6] as a novel method to obtain a summary measure from the probe level data for Affymetrix mRNA arrays. The RMA algorithm was shown to outperform other methods for summarizing multiple probe level data into a single gene expression measure [6]. In our study, we took advantage of the probe replication in the Agilent microRNA arrays, and adapted the RMA algorithm to summarize the microRNA Agilent probe level data into a single processed and normalized microRNA signal estimate. We compared different signal processing methods: the adapted RMA method; the AFE-TGS method with normalization to the 75^th ^percentile, as recommended by the vendor; and the AFE-TGS normalized by the quantile method [7,8]. Irizarry et al. (unpublished) compared the performance of different microRNA array platforms and pointed out that background correction might increase the false positive detection of fold changes for low expressed microRNAs. Therefore we tested the RMA method using probe level data with and without background correction. The different signal processing methods were evaluated in terms of their ability to reduce variability between biological replicates.

## Methods

### RNA samples

We used two independent microRNA gene expression data sets. The first (*dat1*) comprises 8 samples obtained in our lab and the second (*dat2*) contains 31 samples obtained from GEO database [9]. The *dat1 *set comprises the microRNA gene expression profiles of bone marrow-derived human mesenchymal stem cells (hMSCs) and human dermal fibroblasts, obtained from 4 independent donors for each tissue. Total RNA was isolated using the miRNeasy kit (Qiagen). 100 ng of each RNA sample were hybridized to Agilent Human microRNA Microarray v2.0 (G4470B, Agilent Technologies). MicroRNA labeling, hybridization and washing were carried out following Agilent's instructions. Images of hybridized microarrays were acquired with a DNA microarray scanner (Agilent G2565BA), and features were extracted using the AFE image analysis tool version A.9.5.3.1 with default protocols and settings [10]. The hMSCs and dermal fibroblasts used in our study have been deposited in the GEO database [9] (accession number GSE19232) and the corresponding raw data can be retrieved from the supplementary file (GSM476577.txt.gz): MSC_rep1, Fib_rep1, MSC_rep2, Fib_rep2, MSC_rep3, Fib_rep3, MSC_rep4 and Fib_rep4. Since the number of replicates in *dat1 *might be too low to provide compelling evidence, we also analyzed a larger data set (*dat2*), also hybridized to the Agilent Human microRNA microarray v2.0 (G4470B, Agilent Technologies). *dat2 *was selected from the raw data deposited in the supplementary file of the GEO GSE16444 series. *dat2 *is made up of 31 samples from stage 4 neuroblastoma patients: 17 from long survivors and 14 from short survivors. As with the hMSC and dermal fibroblast data, the slides used in the GSE16444 series were scanned with an Agilent G2565BA scanner according to the microRNA Microarray System protocol, and the raw data were obtained with the Agilent Feature Extraction software v. 9.5.3.1 (Agilent Technologies).

### Agilent microRNA microarray

Agilent microRNA assays integrate eight individual microarrays on a single glass slide. Each microarray includes approximately 15 k features containing probes sourced from the *miRBASE *public database [11]. The probes are 60-mer oligonucleotides directly synthesized on the array. In this study we used Human microRNA microarray v2.0, which contains 723 human and 76 human viral microRNAs, each replicated 16 times. 362 microRNAs are interrogated by 2 different oligonucleotides, 45 microRNAs by 3, and 390 microRNAs by 4. Only 2 microRNAs are interrogated by a single oligonucleotide. The array also contains a set of positive and negative controls that are replicated a variety of times. Some of the positive control probes target non-microRNA human RNAs. Each of these targets was interrogated with 4 different probes, which are repeated 5 times. The signals from these positive controls can be bright or dim depending on the sample, and according to Agilent they do not behave consistently enough to be used for normalization.

### Agilent total gene signal

The AFE algorithms estimate a single intensity measure for each microRNA, referred to as the total gene signal (TGS). The AFE-TGS is estimated by multiplying the *total probe signal *by the number of probes per gene. The *total probe signal *is the robust average of all the background-subtracted signals for each replicated probe multiplied by the total number of probe replicates. Usually the background signal is the sum of the median local background signal plus the spatial detrending surface value computed by AFE, which estimates the noise due to a systematic gradient on the array.

### Signal Processing

All the methods used in the study were implemented in R [12] using functions and packages collected in the Bioconductor project [13] as well as custom written routines. Agilent microRNA microarrays interrogate each microRNA with multiple probe sets. The statistical inference requires a processed signal, which is an estimate of the expression measure for every microRNA that can be normalized between arrays. We considered 4 processed signals: a) the AFE-TGS normalized to the 75^th ^percentile (*nor75*); b) the AFE-TGS normalized by the quantile method (*norQ*); c) the adapted RMA algorithm using a background-corrected signal based on the exponential-normal convolution model [6] (*norRMAbg*); and d) the RMA method without background correction (*norRMA*). Negative values in the AFE-TGS were converted into positive signals by adding the quantity |min (AFE-TGS)| + 2 before log transformation. The processed *nor75 signal *was obtained for every array by dividing the AFE-TGS by the 75th percentile of the signal for that particular array. This guarantees that the adjusted signals will all have a 75^th ^percentile equal to 1. The reason for using the 75^th ^percentile rather than other statistical measures such as the mean is to diminish the possible influence of outliers. The median could be used instead, but if we assume that about half of the genes will not show any significant expression, the 75th percentile will represent the median of the remaining 50% that are expressed. The *norQ *was obtained by using the *normalizeBetweenArrays *function from the Bioconductor *limma *package [14]. *norRMAbg *and *norRMA *estimate the expression of a given microRNA from all the probe measures for that microRNA. The RMA algorithm was applied in the following sequential steps. For *norRMAbg *only, the raw mean signal was first background corrected by the exponential + normal convolution model [6], using the *rma.background.correct *function of the Bioconductor *preprocessCore *package [15]. *norRMAbg *and *norRMA *signals were then normalized between arrays by quantile normalization using the *normalizeBetweenArrays *function [13]. The signals were log 2 transformed, and the median of the replicated probes was obtained, normally yielding 2, 3 or 4 different measures (probe level data) for each microRNA; these measures were summarized into a single microRNA measure with the *rma_c_complete_copy *function of the *affy *package [16]. For each feature, the RMA estimates a unique signal by fitting a linear model that takes into account the probe effect. The estimates in the linear model are obtained using the median polish algorithm.

## Results and Discussion

**Figure 1:**
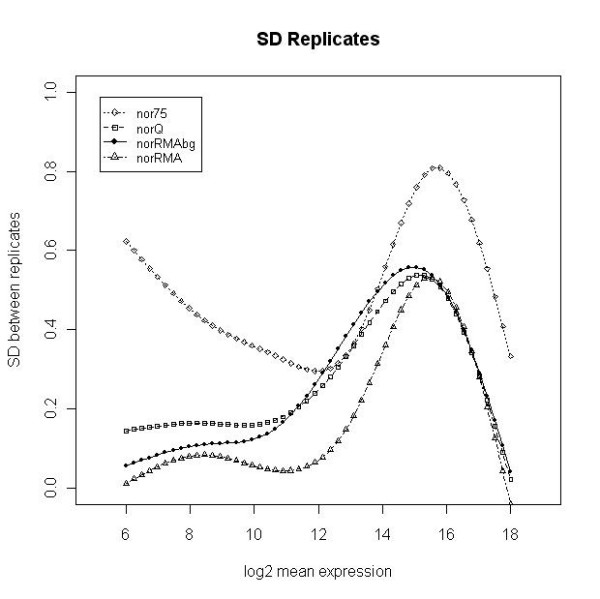
**Signal variability for dat1**. Smooth curves fitted to the scatter plots of SD values for biological replicates against the average expression of each gene (log2 scale) in the *dat1 *data set (8 arrays). Curves were fitted using natural cubic splines with 5 knots.

**Figure 2:**
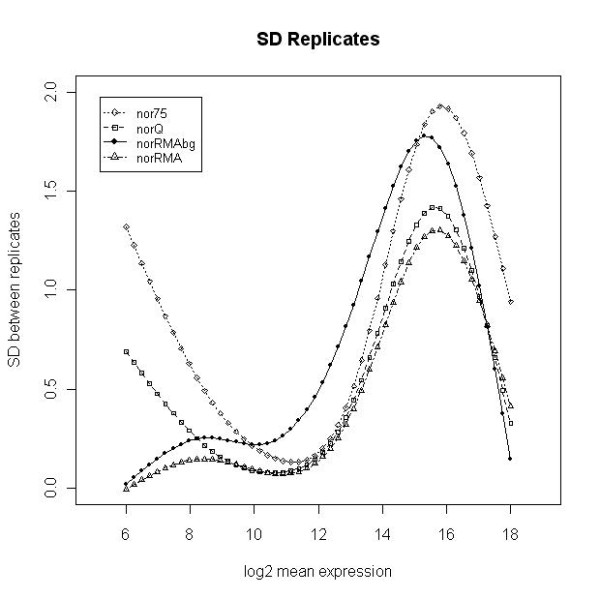
**Signal variability for dat2**. Smooth curves fitted to the scatter plots of SD values for biological replicates against the average expression of each gene (log2 scale) in the *dat2 *data set (GEO accession number GSE16444, 31 arrays). Curves were fitted using natural cubic splines with 5 knots.

**Figure 3:**
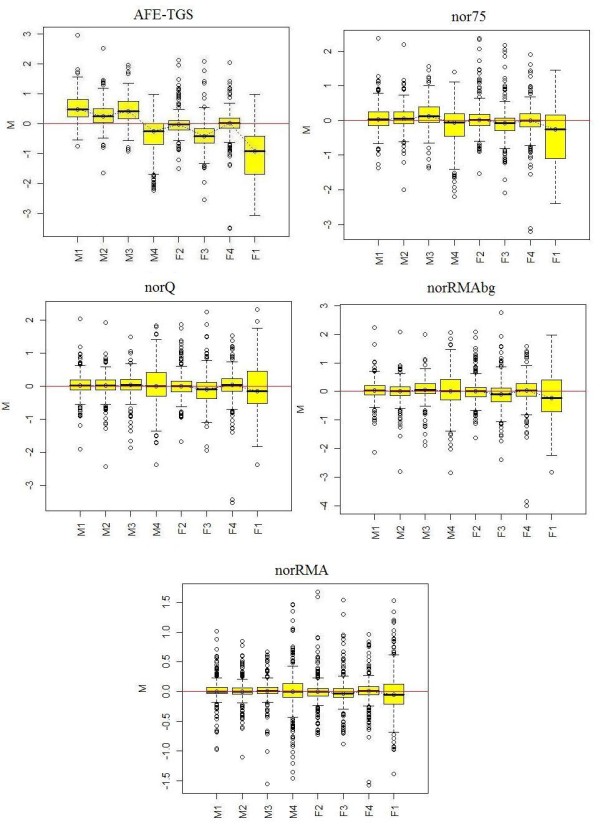
**Relative log expression boxplots for *dat1***. RLE plots (described in the text) for the total gene signals estimated by different methods. *AFE-TGS*, AFE-TGS without normalization between arrays. *nor75*, the AFE-TGS normalized to the 75^th ^percentile. *norQ*, AFE-TGS normalized by quantiles. *norRMAbg*, total gene signal estimated by the RMA algorithm using background-corrected probe-level data. *norRMA*, total gene signal estimated by the RMA algorithm using raw probe-level data without background correction. *M*, hMSC samples; *F*, human dermal fibroblast samples.

We compared four methods for obtaining a processed microRNA signal that can be used for statistical microarray data analysis. The four processed signals analyzed were a) the total gene signal estimated by AFE (AFE-TGS) and normalized to the 75^th ^percentile (*nor75*), as recommended by the vendor; b) the AFE-TGS normalized by the quantile method [7,8] (*norQ*); c) the total gene signal estimated by the RMA algorithm [6] using background-corrected data (*norRMAbg*); and d) the total gene signal estimated by the RMA algorithm using the raw probe-level data without background correction (*norRMA*). Both *norRMAbg *and *norRMA *incorporate the quantile normalization approach [7,8]. The goal of the study was not to compare different normalization methods, but rather to compare different methods for obtaining a summarized gene signal based on multiple probe level data, in this case the AFE-TGS method provided by the vendor and the RMA method, which can be used with or without background correction. Once a total gene signal was obtained, we used the quantile method to normalize between arrays, since this is one of the most robust methods for normalizing between microRNA arrays according to the literature [4,5]. We did not consider other methods because our goal was not to compare normalization methods, but to obtain a summarized measure of microRNA expression from multiple probe level data. For the AFE-TGS we also used the 75% percentile, as this is one of the methods recommended by the vendor. The different signal pre-processing methods were evaluated for their ability to reduce the variability between biological replicates in the two data sets. We computed the SD of the log2 expression values for every feature across the biological replicates. We then used natural cubic splines (5 knots) to fit curves to the scatter plot of the SD values against the average expression values. Similar conclusions can be drawn from both data sets. The *nor75 *signal has the largest variability among replicates across almost all intensity ranges for *dat1 *(figure 1), although for *dat2 *the *norRMAbg *has slightly larger SD values for medium intensity values (figure 2). The *norQ *and the *norRMAbg *signals show approximately the same variability for *dat1 *(figure 1). The larger SD for medium intensity values obtained with *norRMAbg *in *dat2 *is due to the background correction. Interestingly, the SD values obtained with uncorrected and background-corrected signals in the RMA method follow similar profiles, but the SD values are larger for the background-corrected signals. The same pattern can be seen in *dat1*, but in this data set the background correction seems to increase signal variability to a much lesser extent. The *norRMA *signal has the smallest variability for both data sets. For high intensity values, the *norQ*, *norRMA *and *norRMAbg *curves overlap, especially for the *dat1 *data set, suggesting that the choice of one method over another might have more influence on the detection of low-expressed microRNAs. After obtaining the normalized signals from *dat1*, we removed the positive and negative controls and the microRNA genes that were not expressed in either of the two experimental groups (hMSCs and fibroblasts) according to the *IsGeneDetected *flag provided by AFE algorithms. After this filtering step only 284 out of 799 microRNA genes were retained, as considered to be expressed in at least one experimental group. To study the overall effect of the different pre-processing methods we generated relative log expression (RLE) boxplots for the 284 expressed microRNAs (figure 3). The RLE boxplots plot the differences between the processed microRNA signals from each array and the median of the same signals across all the arrays [17]. The RLE boxplots should be centered on zero and all of them should have approximately the same dispersion. The RLE of the non-normalized AFE-TGS shows a high dispersion between arrays, and it is also evident that the hMSC samples have an overall higher intensity than the fibroblasts. The 75% percentile normalization (*nor75*) tends to push the sample medians towards zero, but slight differences between mesenchymal and fibroblast samples can still be observed. After normalizing the AFE-TGS by quantiles (*norQ*) all the boxplots are centered on zero and have a similar dispersion. Boxplots generated with the RMA algorithm data also centered on zero. This is not surprising since the RMA method incorporates the quantile algorithm, but the RMA algorithm seems to further reduce the variability of the signals in comparison with the *norQ *signal. The variability was especially reduced for the RMA method with no background correction (*norRMA*). This effect was also pointed out by Irizarry et al. (unpublished).

## Conclusions

Taking advantage of the probe replication in Agilent microRNA arrays, we have adapted the RMA algorithm to obtain a summary microRNA signal that takes into account the probe affinity effect. In addition, since there is evidence that omission of background correction might yield less variable results than methods that use background-corrected signals (Irizarry et al. unpublished), we applied the RMA algorithm to uncorrected and background-corrected signals, both normalized by quantiles [7,8]. The RMA of the non background-corrected signal showed lower variability between replicates than either the RMA of the background-corrected signal or the quantile-normalized AFE-TGS [7,8], especially for the *dat1 *data set. For the *dat2 *data set the RMA of the non background-corrected signal still showed lower variability than the AFE-TGS normalized by quantiles, although the differences in this case were smaller. The high signal variability obtained for *dat2 *with background correction in the RMA method suggests that an RMA method using a non background corrected signal might be preferable. The RMA of the non background-corrected signal and the AFE-TGS normalized by quantiles were almost equally precise, but the RMA seems to produce signals of lower variability at low intensity values. The use of the RMA algorithm with an uncorrected signal might thus be advantageous for the detection of low expressed genes. Finally, the AFE-TGS normalized to the 75% percentile showed the highest signal variability of all methods tested, indicating that quantile normalization yields lower signal variability than the method recommended by the vendor.

## Competing interests

The authors declare that they have no competing interests.

## Authors' contributions

PLR did the analysis and wrote the paper. MAG processed and obtained the biological samples. SC and AD did the microRNA hybridizations and image analysis, and generated of the Feature Extraction data files. RAI supervised the study. All the authors read and approved the final manuscript.
